# A Thermal Actuated Bistable Structure for Generating On-Chip Shock Loads

**DOI:** 10.3390/mi13040569

**Published:** 2022-04-02

**Authors:** Runze Yu, Dacheng Zhang

**Affiliations:** Institute of Microelectronics, Peking University, Beijing 100871, China; yrzdx@pku.edu.cn

**Keywords:** bistable structure, thermal drive, shock test

## Abstract

In this paper, we propose a bistable shock structure based on the thermal actuation principle, which overcomes the response time limitation of heating and cooling in typical thermal actuators and enables a rapid release of energy. Thus, force with a steep rising edge can be applied on a target. Using a bistable shock structure to generate on-chip shock loads, we propose an automated and resettable method for shock testing of microstructures. We characterize the microscale shock process by high-speed camera and finite element simulation (FEM). The method can simulate the dynamic response of key structures in MEMS devices under mechanical shock conditions, and therefore, can be used to evaluate shock fracture strength of microstructures.

## 1. Introduction

**Figure 1:**
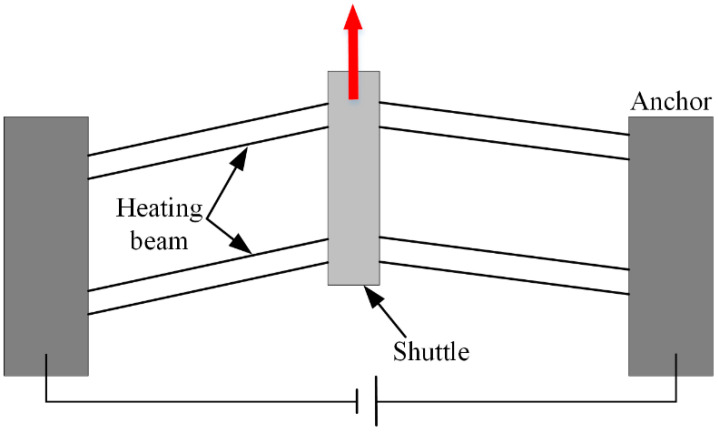
Diagram of the V-shaped thermal actuator.

Microactuators are one of the key technologies of a MEMS system, and a common method is the thermal drive principle [1,2,3], which realizes the conversion of electrical and mechanical energy as a result of the thermal buckling of structures. Commonly used thermal actuators are bimorph actuators [4], U-shaped actuators [5], and V-shaped actuators [6]. Figure 1 shows a diagram of a V-shaped thermal actuator. When an electric current is passed through a V-shaped heating beam, the beam generates ohmic heating and elongates in length. Because the two ends are restrained, the lengthening of the beam causes buckling and the shuttle in the middle is moved. A V-shaped thermal actuator is a classic model that has been extensively studied [7,8,9]. Although a typical thermal actuator can generate a large output displacement and output force, its response speed is low. Because heat conduction and expansion are not instantaneous processes, it is difficult for a thermal actuator to apply a shock force with a steep rising edge to a target. The time constant of a thermal actuator reflects its dynamic responsiveness and is determined by the heating/cooling time.

Response speed is usually affected by the feature size, input power, and heat transfer properties of a material. Lijie Li and Deepak Uttamchandani [10] researched a 1.8 mm × 0.6 mm × 0.1 mm bimorph in-plane actuator and the electrothermal rise time for the whole actuator was 17.3 ms. T. Seki et al. [11] made a 2.5 mm × 0.5 mm × 0.02 mm actuator with silicon and the response time was 5 ms at 27 V/25 mA input power. Li-Sheng Zheng and Michael S.-C. Lu [12] fabricated a 300 μm × 17 μm × 5 μm thermal actuator in a conventional CMOS process. Their actuator achieved an out-of-plane displacement of 24 μm at an applied power of 17 mW and a thermal time constant of 0.24 ms. Generally, reducing the size improves the response speed; however, a small size limits the output capability of thermal actuators. A small actuator structure cannot be used to drive a movable structure, and therefore, it is used more in the MEMS relay. If the elastic potential energy caused by thermal expansion could be stored and released instantly through a trigger device, this problem would be solved. Mechanical bistable systems that typically rely on strain energy storage to gain bistable behavior are suitable for this [13]. Some researchers have utilized the snap-through effect of a fixed-fixed beam buckled under compressive residual stress [14,15]. Other researchers have used a mixture of rigid-body and compliant joints to achieve bistable behavior [16]. In this paper, a bistable structure is formed by applying prestress to a beam through the thermal drive principle and, in this way, the quick response and shock function of the thermal actuator is realized.

Excitingly, this structure enables in situ shock tests to be directly loaded on the microstructure. Most on-chip tests study microstructure tensile fracture by probe [17], thermal actuator [18], and electrostatic actuator [19], but an on-chip shock test is a new concept. In recent years, although there has been a lot of studies on mechanical shock in MEMS, most of them have investigated the shock reliability of the device through shock testers. A drop tower tester [20] is designed to drop a device from a high place and the acceleration environment is due to the impact. A Hopkinson bar [20,21,22,23] can apply stress waves to a sample to create accelerations in excess of 10,000 g. In ballistics testing [24,25], a projectile containing a device is launched to impact a target and produce an impact. The individual damage mechanisms of devices are hard to analyze because of the complexity of the devices. It is difficult to truly characterize a microstructure’s shock fracture strength by such device-level tests. For MEMS devices that work in mechanical shock conditions, obtaining a structure’s shock fracture strength is helpful to guide the design of support and protection structures. Furthermore, the collision behavior between microstructures is also worth studying. A typical example is a MEMS inertia switch. M. I. Younis et al. [26] pointed out that when the movable microstructures collided with other components or substrates, they cracked due to severe contact stress. Because of the above issues, it is difficult for existing test methods to directly apply shock load to a microstructure. The bistable structure proposed in this paper provides a means for studying these issues.

In this paper, first, in Section 2, we introduce the basic principle and working process of the bistable shock structure and provide a mechanical analysis. Using the bistable shock structure, we design an automated and resettable on-chip shock tester. The design details of the tester and its modified version are shown. In Section 3, we present the process flow used to manufacture the tester and, in Section 4, we use the tester to perform shock tensile tests on the fixed-fixed beam samples. The experimental results are demonstrated by FEM and a high-speed camera.

## 2. Principle and Design

### 2.1. Bistable Shock Structure

**Figure 2:**
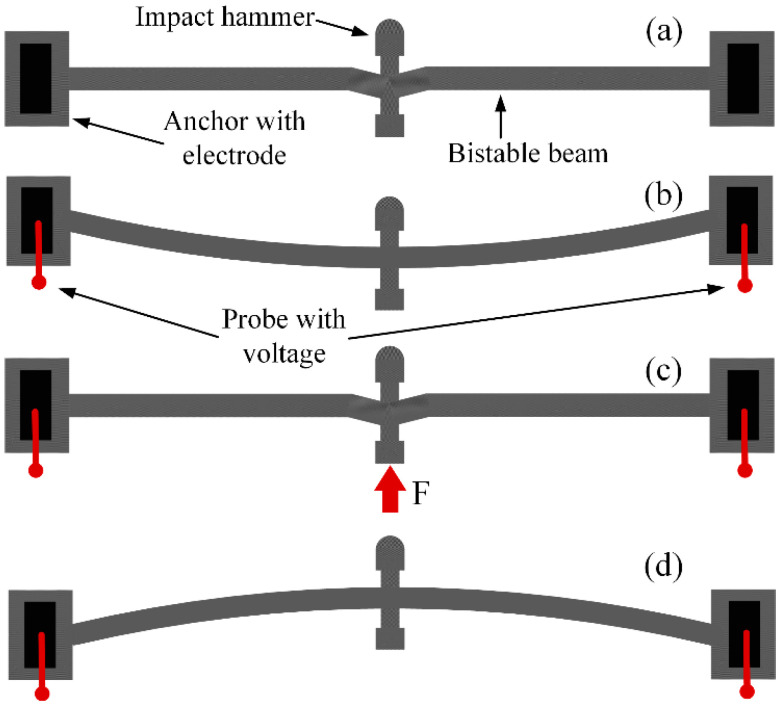
Working process of bistable beam. (**a**) Initial state. (**c**) Critical point for steady-state switching. When loading voltage, it has two stable states (**b**,**d**) because of thermal expansion. The external force F helps to change (**b**) to (**d**).

The movable range of a shock structure is small because of the dimensional limit of MEMS devices. To realize the high-speed shock process, a shock structure is required to store a large amount of energy and to release it in a short time. Figure 2 presents a schematic diagram of a bistable beam and its working principle; both sides of the bistable beam are fixed on anchors, and electrodes are patterned on the anchors. A shock hammer is designed at the middle of the bistable beam. The beam has two different stable positions after the voltage is loaded on electrodes, as shown in Figure 2b,d. A small inclination angle is designed at the joint of the beam and the shock hammer, and therefore, the force generated by the thermal expansion has a backward component, and the bistable beam is bent backward. After the bistable beam reaches the thermal steady state, an external force F is applied to the tail of the shock hammer and the bistable beam is compressed by F, which is an energy storage process. When the bistable beam reaches the critical point, shown in Figure 2c, the maximum energy storage is reached and energy is released immediately, which provides a high shock hammer with high acceleration.

**Figure 3:**
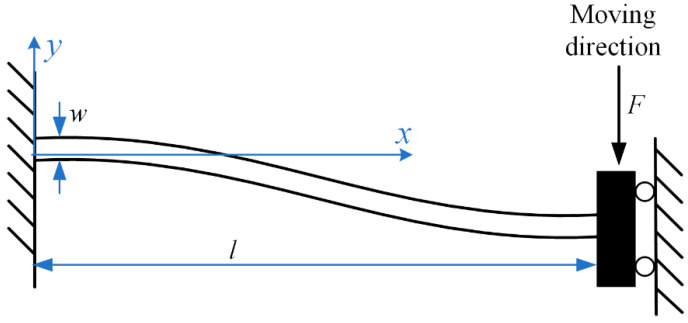
The deformation of a fixed-guided beam. It is half of the bistable beam.

The size of the bistable beam determines how much energy it stores and the maximum shock load of the shock tester. A bistable beam is considered to be pushed by the lateral force *F* and compressed by the axial force *F_y_* during the energy storage process. Because the beam extends along its length and because of thermal expansion, we ignore the change in the cross-sectional area. The mechanical model can be simplified as a fixed-fixed beam subjected to a concentrated load in the middle. Because a bistable beam is a symmetric structure, we take half of the structure for analysis. It is a beam with one end fixed and one end guided, and the deformation is shown in Figure 3. The deflection curve can be expressed as:

(1) $$y=-\frac{F}{12EI}(3lx^{2}-2x^{3}),0\leq x\leq l$$

The deflection of guided end is:

(2) $$y=-\frac{Fl^{3}}{12EI}$$

The effective stiffness of fixed-guided beam is:

(3) $$k=-\frac{F}{y}=\frac{12EI}{l^{3}}=\frac{Ehw^{3}}{l^{3}}$$

where *E* is Young’s modulus and *l*, *w*, *t* are the length, width, and height of the beam, respectively. In small-deflection condition, the elastic potential energy caused by *F* is:

(4) $$E_{t}=2\times \frac{1}{2}kd^{2}=\frac{Ehw^{3}d^{2}}{l^{3}}$$

where *d* is the deflection in the middle of bistable beam, which is equal to the displacement of the shock hammer in Figure 2b.

Due to the axial restraint at the guided end of the beam, the beam is actually extended. Therefore, the deformation energy caused by the axial extension also needs to be considered. For the fixed-guided beam, when there is no axial restraint at the guided end, the bending moment does not cause the beam axis to elongate or shorten, but does cause the guided end to “retract” toward the fixed end. The axial displacement caused by the bending moment is:

(5) $$\delta_{1}\left(x\right)={\int_{0}^{x}-\frac{1}{2}\left(\frac{dy}{dx}\right)}^{2}dx=-\frac{F^{2}}{8E^{2}I^{2}}\left(\frac{l^{3}x^{3}}{3}-\frac{lx^{4}}{2}+\frac{x^{5}}{5}\right)$$

Due to the axial restraint of the guided end, there is no actual “retraction”. Therefore, the axial force causes the extension of the axis to be:

(6) $$\delta \left(x\right)=\frac{0-\delta_{1}(l)}{l}x=\frac{F^{2}l^{4}}{240E^{2}I^{2}}x$$

The axial deformation energy is:

(7) $$E_{y}=2\times \frac{1}{2}EA{\int_{0}^{l}\left(\frac{d\delta }{dx}\right)}^{2}dx=\frac{9Ewhd^{4}}{50l^{3}}$$

The total elastic potential energy stored in a bistable beam is *E_t_* + *E_y_*. For bending deformation and tensile deformation, the force provided by a bistable beam to a shock hammer contains the first order and the third order of the deflection, respectively. The third order item caused by the axial force leads to the nonlinearity of stiffness. As a nonlinear structure, the advantage is that more energy can be stored within the allowable stress range.

**Figure 4:**
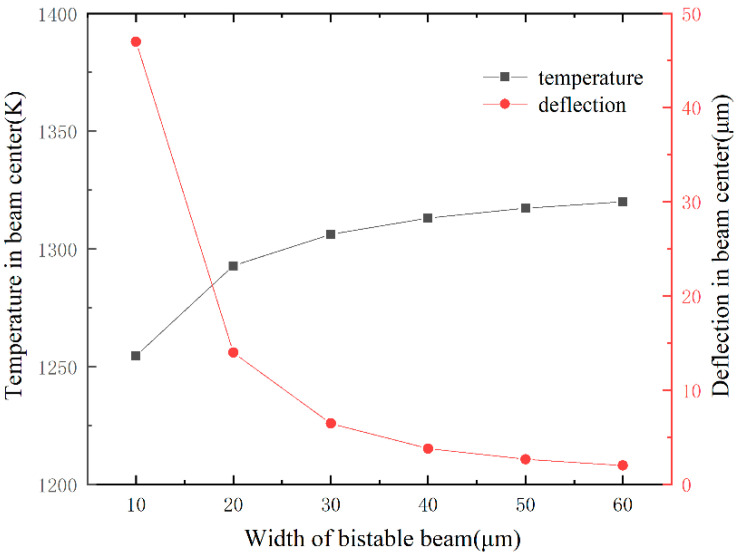
The relationship of the beam’s width to the beam’s temperature and the maximum deflection of the center (length of the beam is 1000 μm).

**Figure 5:**
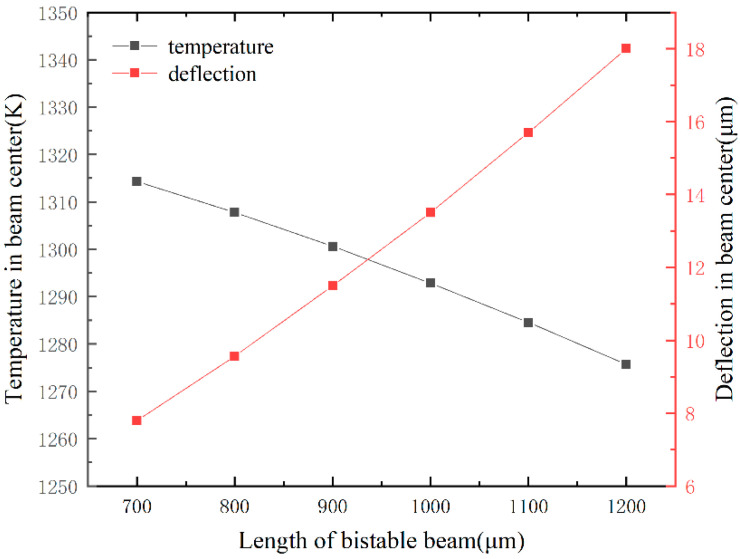
The relationship of the beam’s length to the beam’s temperature and the maximum deflection of the center (width of the beam is 20 μm).

To guide the size design, we studied the relationship between size and the center deflection by FEM. The simulation of the bistable beam is similar to the V-shaped thermal drive beam. We consider the steady state modeling of a bistable beam and do not consider the effects of free convection and radiation [27], using ANSYS element SOLID98. A constant voltage of 5 V is applied on the bottom surfaces of anchors as boundary conditions. Due to the better heat dissipation of the metal tray, the substrate is held at near ambient temperature, and room temperature (300 K) is applied on the bottom surfaces of the anchors as boundary conditions. The beam mainly dissipates heat through the substrate [28]. The simulation parameters about material properties and convective heat transfer coefficient are shown in Table 1. Figure 4 shows the relationship of the beam’s width to the beam’s temperature and the maximum deflection of the center when the length of the bistable beam is 1000 μm. When the length and voltage are constant, the wider the bistable beam, the smaller the beam resistance and more ohmic heating is generated, and therefore, the temperature of the beam increases with an increase in the width. A wider beam has greater stiffness, therefore, the maximum deflection of the center is less when compressed by the axial force. Movable bulk silicon structures are attached to glass substrates. Considering the influence of heat radiation and convection on the glass substrate, the temperature of the bistable beam should not exceed 1300 K. Figure 5 shows the influence of beam’s length on working capacity when the width of the bistable beam is 20 μm. The maximum temperature is negatively correlated with the beam’s length. Because the length increases, the resistance increases, and therefore, the power becomes lower under constant voltage conditions, and the heat dissipation area is also increased. The maximum center deflection is positively correlated with beam length because the longer the beam, the greater the thermal expansion [29]. Table 2 shows designed dimensions of the bistable structure. Through simulation, for a bistable beam of the designed size, the loading force F is proportional to the displacement, consistent with the literature report [30]. The maximum deflection of the bistable beam reaches 13.5 μm, and the loading force required for the bistable structure to reach the critical point is 2.77 mN. The maximum speed the shock hammer can reach is 20 m/s.

### 2.2. Shock Tester

**Figure 6:**
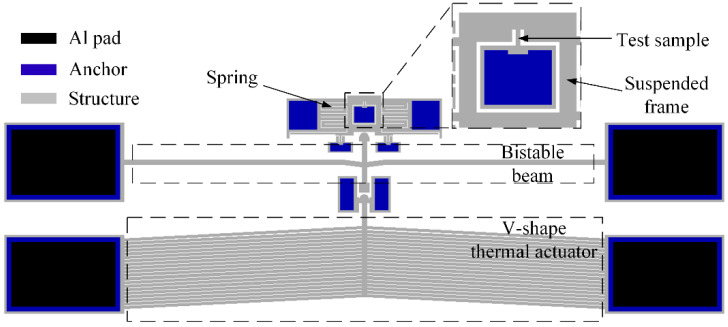
Schematic diagram of the Type I shock tester based on a bistable beam. The anchors on both sides of the tail of the shock hammer are scales. The external force F was provided by the V-shape thermal actuator.

Based on the created bistable beam, the schematic diagram of the entire tester is shown in Figure 6. This is the Type I tester. The test sample and tester were manufactured under the same process, and therefore, they could be effectively integrated. The sample for the shock tensile test is shown. One end of the sample is connected to an anchor, and the other end is connected to a suspension frame. The suspension frame is a reversing component that converts push into pull. The anchors on both sides of the tail of the shock hammer are etched into scales, and therefore, the displacement of the shock hammer during energy storage could be read by the scale under optical microscope. In this tester, the external drive to push the impact hammer should generate a large displacement and force output. The high output force and displacement of thermal actuators is suited to switch bistable structures [28], therefore, the external force F is provided by the V-shaped thermal actuator, which can realize automation and repeatability loading. To improve the driving ability of the thermal actuator, we designed the structure composed of multiple sets of thermal arms. Its key dimensions are also shown in Table 1, which meets the driving requirements of the created bistable beam.

**Figure 7:**
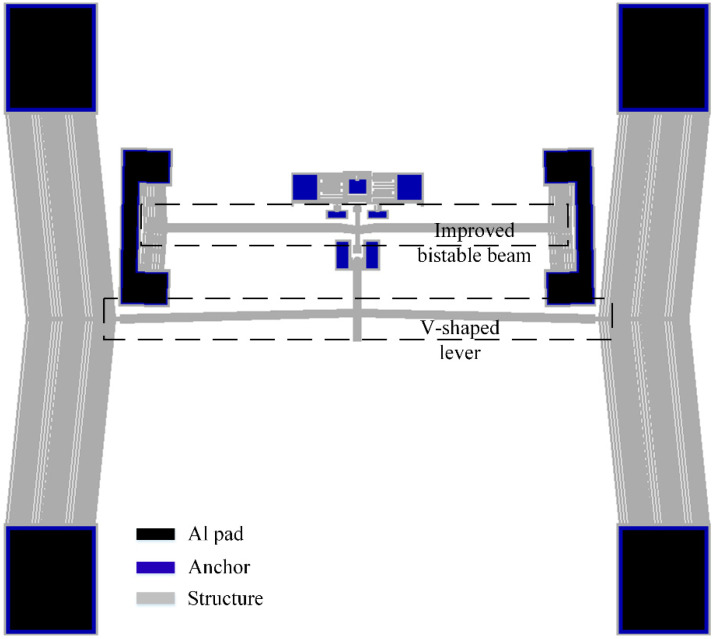
Type II tester with cascaded V-shaped thermal actuators.

A cascaded thermal actuator system can be used to amplify output [35]. To improve the output capability of the bistable shock beam, we made improvements to the tester and designed the Type II tester. V-shaped thermal actuators are designed at both ends of the bistable beam, as shown in Figure 7. This can further increase the deformation of the bistable beam. Correspondingly, we modified the thermal actuator used to push the shock hammer. We increased its displacement output by a V-shaped lever with two V-shaped thermal actuators cascaded on both sides. The dimensions of the V-shaped thermal actuator are unchanged. Through FEM, the maximum deformation of the improved bistable beam at 5 V is 39.2 μm. The maximum deformation is increased by 2.9 times as compared with the previous bistable beam. Integrating the loading force on the displacement of the shock hammer, it can be obtained that the bistable beam stores six times more elastic potential energy than before. The dimensions of the modified structure are shown in Table 3.

## 3. Fabrication

**Figure 8:**
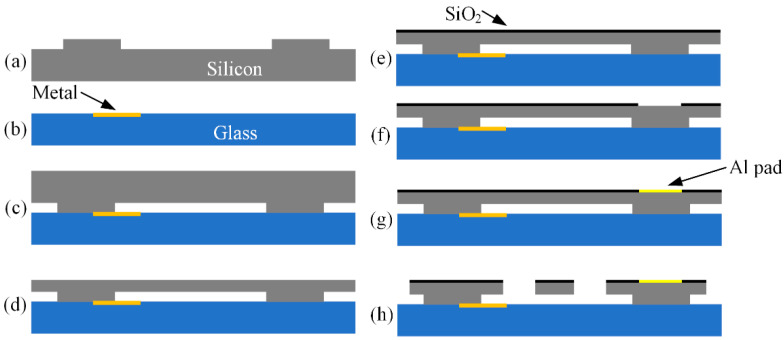
The fabrication process flow of the shock tester: (**a**) Etching for anchor; (**b**) forming Ti/Pt/Au layer; (**c**) Si-glass anodic bonding; (**d**) bulk silicon thinning; (**e**) PECVD SiO_2_; (**f**) define electrode area; (**g**) PVD Al; (**h**) DRIE etching.

The process of manufacturing the tester in this paper is improved on the basis of our previous work [17]. The fabrication process is shown in Figure 8. Because thermal actuators need to generate large displacement outputs through thermal expansion, the electrodes need to be able to support enough current. It is difficult for the electrodes and wires formed on the glass to meet the requirements, because of the electromigration due to their small thickness. We used *n*-type (100) silicon wafers as the structural material. To increase power, the resistivity of silicon was chosen to be 0.001–0.003 Ω cm. The thickness of the anchor was 5 μm. At the same time, the Ti/Pt/Au pattern was made on the glass using the lift-off process. It is used to prevent the footing effect in deep reactive ion etching (DRIE). Footing effect refers to when the etching gas encounters the insulating layer (SiO_2_) and it continues to etch laterally near the interface. The metal can draw away the charge collected on the insulating layer. After the silicon-glass anode was bonded, the silicon wafer was etched to the designed thickness with KOH solution. When the movable structure was formed, the etching depth of the bulk silicon was often about 70 μm, and it was difficult for the ordinary thickness of photoresist to achieve protection. Thick photoresist is often prone to errors in photolithography. For this reason, a layer of silicon dioxide was deposited by PECVD as an etching mask. Then, electrodes were formed on the surface of the structure by a lift-off process. Finally, movable suspension structures were formed by DRIE.

## 4. Experimental Results

**Figure 9:**
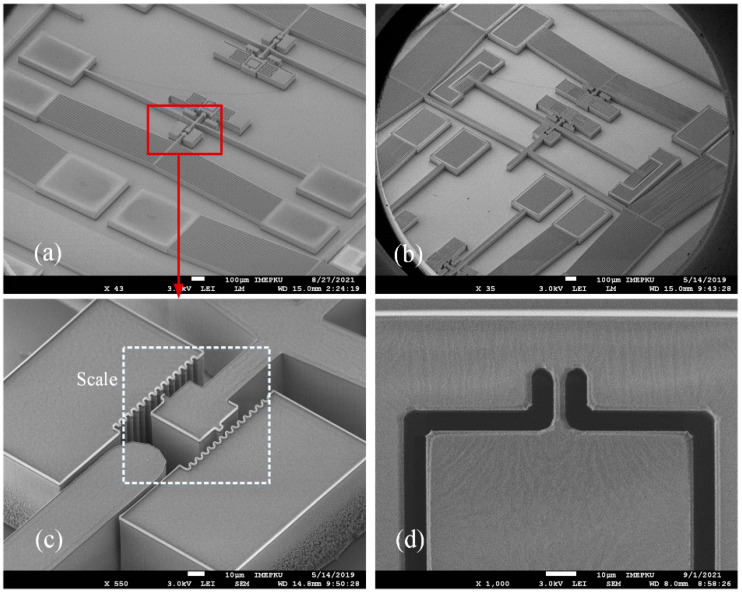
SEM images of tester: (**a**) Type I tester; (**b**) Type II tester; (**c**) scale that can be used to obtain displacement; (**d**) test sample.

The SEM images of the Type I and Type II testers are shown in Figure 9. We tested on the MM6000 probe station. After the probes contacted the electrodes at both ends of the bistable beam, we adjusted the voltage. Usually, we started with a voltage of 1 V. The displacement of the shock hammer could be observed through the optical microscope on the probe station, and we slowly increased the voltage to set displacement. Then, we applied a voltage across the V-shaped thermal actuator, slowly increasing the voltage until the hammer was pushed past the critical point. This process could be repeated, and by changing the initial displacement of the shock hammer, the dynamic response of the sample under different shock energies coud be observed.

**Figure 10:**
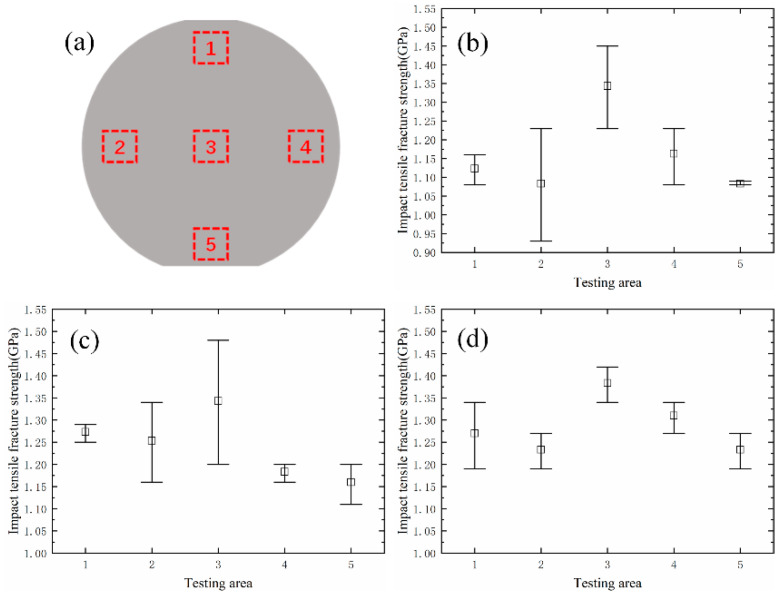
(**a**) Test area partition. Regional statistical results of samples with different widths of: (**b**) 3 μm; (**c**) 4 μm; (**d**) 5 μm.

**Figure 11:**
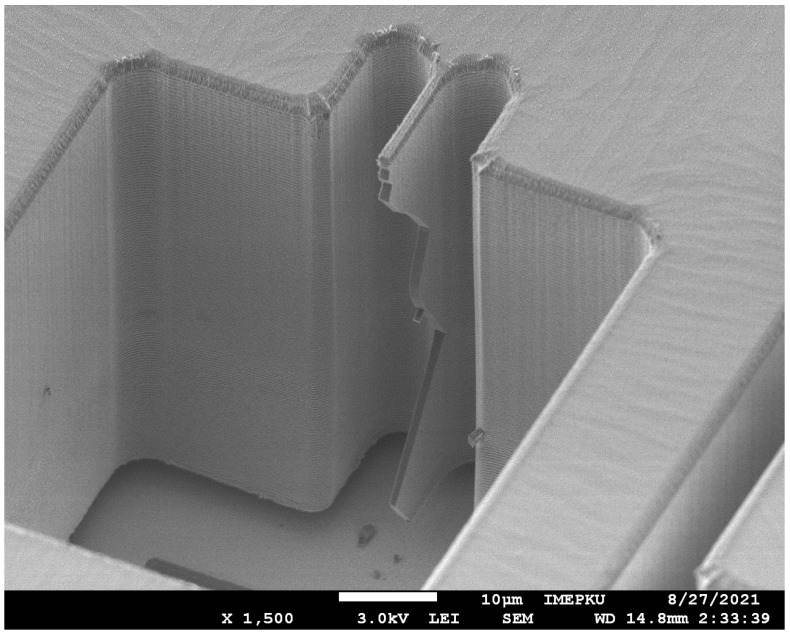
SEM image of a sample cross-section after fracture.

**Figure 12:**
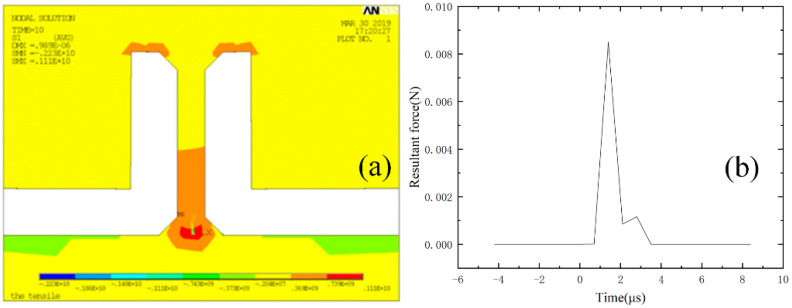
FEM results when the displacement of shock hammer is 10 μm: (**a**) Stress distribution diagram of the test sample when shock happens; (**b**) resultant force on contact surface.

To reflect the distribution of the fracture strength parameter on the wafer, the entire bonded wafer was tested in the partition, as shown in Figure 10a. Each partition was tested multiple times to ensure the repeatability of the test results. Figure 10 shows the regional statistical results of the test samples of different sizes. The fracture strength of the test sample was obtained by FEM. It can be seen in Figure 11 that the test sample broke in the root area. As shown in Figure 12a, the stress concentration point of the sample was also in the root area. The experimental results were consistent with the FEM results. We achieved the resultant force on the contact surface. The shock pulse force worked within 4 μs.

**Figure 13:**
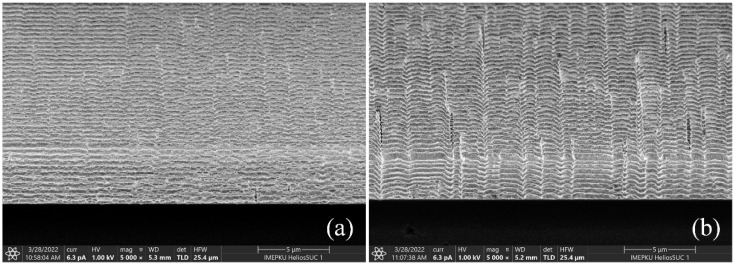
The sidewall etching effect of different areas on the wafer: (**a**) Center area; (**b**) edge area.

The shock tensile fracture strength is between 0.93 and 1.48 GPa, which is higher than the quasi-static tensile results [17]. The fracture surfaces are flat and show a tear texture, which is consistent with the characteristic of brittle materials. The sample primarily broke aligned with the (111) crystal plane, which was due to the greatest spacing between the crystal planes and the lowest surface energy to resist fracture [36]. In terms of uniformity across an individual wafer, the shock fracture strength of the central region was higher than that of the edge region of the wafer. This conclusion is consistent with the actual etching process: the electromagnetic environment at the edge of the wafer is more complex, and the plasma concentration of the excitation source is different, which leads to the lower quality of the DRIE at the edge than the central region. The sidewall etching effect of different areas on the wafer is shown in Figure 13.

**Figure 14:**
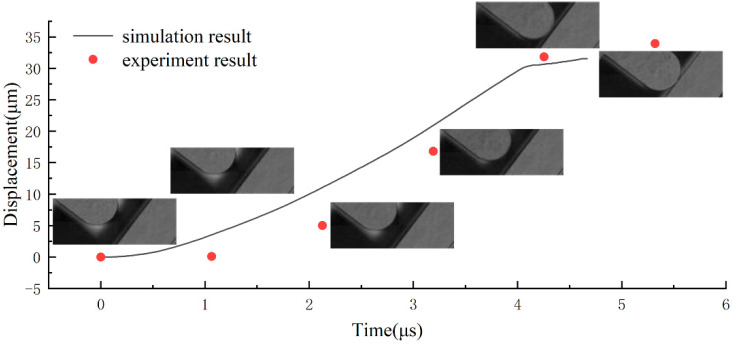
High-speed camera recorded a shock process of the tester. The simulation results are compared with the high-speed camera results.

The shock hammer speed, as the initial input of the simulation, determines the accuracy of the test results. To validate the simulation model in the test, we used a high-speed PhantomV2512 camera to record a shock process and calculated the speed of the shock hammer. PhantomV2512 was used for imaging at a speed of 940,000 frames per second. The experimental results obtained from the high-speed camera and the simulation results are compared in Figure 14. The actual velocities are approximately the same as those obtained from FEM, which validates the simulation model.

## 5. Conclusions

This work provides a proposed solution to the contradiction between the response speed and output capability of typical thermal actuators. It enables a “quick launch” of the thermal actuator. Using the created thermal bistable structure, a tester and its modified version are designed that can generate on-chip shock loads. The tester allows direct high-speed shock tests of microstructures. By controlling the load voltage, the experiment is automated. Furthermore, it is resettable, and therefore shock test can be performed at different shock energies. A high-speed camera verifies the accuracy of the simulation model.

In the future work, we will consider the control of heat dissipation of the tester. We will also further develop the complete energy conversion model and the crack model of etching.

## Figures and Tables

**Table 1:** Conditions of FEM simulation.

Parameter	Value
Young modulus (E)	169 GPa [31]
Poisson ratio	0.28 [31]
Density	2330 kg/m^3^
Thermal expansion	2.6 × 10^−6^ [32]
Thermal conductivity	150 W m^−1^ K^−1^ [33]
Resistivity	0.002 Ω cm
Specific heat	730 J kg^−1^ K^−1^ [34]
Convective heat transfer coefficient	100 W m^−2^ K^−1^ [28]

**Table 2:** The design dimensions of the tester.

Dimension	Value
Length of bistable beam	1000 μm
Width of bistable beam	20 μm
Length of thermal beam	1000 μm
Width of thermal beam	10 μm
Angle of thermal beam	6°
Number of thermal beams	20
Thickness of structure	60 μm

**Table 3:** The dimensions of the modified structure.

Part	Dimension	Value
Thermal actuator at both ends of the bistable beam	Length Width	200 μm 10 μm
	Angle	6°
	Number	10
V-shaped lever	Length Width	1150 μm 40 μm
	Angle	4°
